# Am I safe? An Interpretative Phenomenological Analysis of Vulnerability as Experienced by Patients With Complications Following Surgery

**DOI:** 10.1177/10497323221136956

**Published:** 2022-11-02

**Authors:** Elizabeth Sutton, Lesley Booth, Mudathir Ibrahim, Peter McCulloch, Mark Sujan, Janet Willars, Nicola Mackintosh

**Affiliations:** 1 Department of Health Sciences, 4488 University of Leicester, Leicester, UK; 2 454738 Cambridge Rare Disease Network, Camrbidge, UK; 3 Nuffield Department of Surgical Sciences, 150584 University of Oxford, Oxford, UK; 4 Department of General Surgery, Maimonides Medical Center, Brooklyn, NY, USA; 5 Human Factors Everywhere Ltd., UK

**Keywords:** patient safety, post-surgical deterioration, vulnerability, patient involvement

## Abstract

Abdominal surgery carries with it risks of complications. Little is known about patients’ experiences of post-surgical deterioration. There is a real need to understand the psychosocial as well as the biological aspects of deterioration in order to improve care and outcomes for patients. Drawing on in-depth interviews with seven abdominal surgery survivors, we present an idiographic account of participants’ experiences, situating their contribution to safety within their personal lived experiences and meaning-making of these episodes of deterioration. Our analysis reveals an overarching group experiential theme of vulnerability in relation to participants’ experiences of complications after abdominal surgery. This encapsulates the uncertainty of the situation all the participants found themselves in, and the nature and seriousness of their health conditions. The extent of participants’ vulnerability is revealed by detailing how they made sense of their experience, how they negotiated feelings of (un)safety drawing on their relationships with family and staff and the legacy of feelings they were left with when their expectations of care (care as imagined) did not meet the reality of their experiences (care as received). The participants’ experiences highlight the power imbalance between patients and professionals in terms of whose knowledge counts within the hospital context. The study reveals the potential for epistemic injustice to arise when patients’ concerns are ignored or dismissed. Our data has implications for designing strategies to enable escalation of care, both in terms of supporting staff to deliver compassionate care, and in strengthening patient and family involvement in rescue processes.

## Introduction

Clinical deterioration following abdominal surgery is both a medical emergency as well as a personal crisis for patients. There is a need to understand the psychosocial as well as the biological aspects of this experience in order to improve care and outcomes for patients.

### Failure to Rescue in Surgery

Patients are at increased risk of mortality following emergency abdominal surgery. The death rate after an emergency laparotomy is 5 times higher than for similar elective surgery (Havens et al., 2015). One reason for this higher death rate is that patients may experience deterioration following surgery which despite monitoring for physiological symptoms, clinicians may sometimes miss, misidentify, or mismanage (Odell et al., 2010).

Failure to rescue, defined as the death of a patient after one or more potentially treatable complications (Silber et al., 1992), acts as a useful surgical quality indicator, given that ‘prompt recognition and treatment of complications is a critical, actionable point during a patient’s postoperative course’ (Portuondo et al., 2019). Over the past two decades, attention has focused on strategies to improve the escalation process including those targeting the ‘afferent limb’, that is, enabling calling for help (e.g. track and trigger systems), those addressing the ‘efferent limb’, that is, enabling prompt response (e.g. rapid response teams and standardised protocols) and quality improvement components, enabling data intelligence and feedback to support change (Winters et al., 2013).

However, despite receiving widespread policy and practice attention, escalation of care in surgery remains highly variable, leading to inconsistencies in outcomes (Johnston et al., 2015), indicative of its nature as a complex ‘wicked problem’ (Rutter et al., 2017). There is a comprehensive evidence base detailing continuing difficulties associated with the escalation process. Contributory factors include: junior staff’s lack of clinical experience; professional deference; boundaries between medical teams; unclear protocols; poor supervision and availability of senior staff and high workloads (Johnston et al., 2015)

Burke et al. (2020) provide a useful review of improvement strategies currently being used to tackle these problems. These include standardised track and trigger systems such as the National Early Warning Score (NEWS2) (Royal College of Physicians, 2017), use of electronic alerting systems and Artificial Intelligence (AI) (Holdsworth et al., 2021; Subbe et al., 2017) to aid recognition and response, communication protocols to bridge professional hierarchies (Mackintosh & Sandall, 2010) and response teams to bridge occupational divides (Carmel & Baker-McClearn, 2012).

### Patient and Family Involvement in Escalation of Care

Patients are increasingly regarded as a ‘smoke alarm’ in identifying problems within the NHS (Bacon, 2010) and research studies have explored the involvement of patients in spotting deterioration and escalating care (Albutt et al., 2020; Mackintosh et al., 2017; McKinney et al., 2021). Patients and families bring tacit knowledge to support the ongoing work of healthcare professionals (O’Hara et al., 2018). Patients and families can act as ‘knowledge brokers’ filling ‘structural holes’ between otherwise interconnected parts of a network (Bishop & Waring, 2019).

Call for Concern (Odell et al., 2010) was introduced to enable patients to directly access critical care outreach (CCO) teams in the event of a concern following transfer from the Intensive Care Unit (ICU) to surgical wards. Contrary to concerns expressed by CCO teams, this intervention did not increase staff workload and was reported to be welcomed by patients and relatives.

Albutt and colleagues explored the use of a method to capture patient-led identification of deterioration and found that patients were willing and able to report information about how well they felt during routine observations (Albutt et al., 2020). McKinney and colleagues (2021) conducted a qualitative systematic review of patient and family involvement in escalating concerns about clinical deterioration. They found that any involvement depended on the capacity and capability of patients to identify deterioration, as many patients were unable to discern a change in their clinical condition. The authors also stressed the importance of factors that influence the empowerment of patients, such as system and process factors and the overall culture of the ward or unit.

A further issue in involving patients in the event of possible deterioration is that patient input may not be given the same credence as that of clinicians. Patient input has been found to lack legitimacy when compared to more ‘objective’ clinical indicators (Entwistle et al., 2010). Indeed, Kid and Carel point out that the very ‘structures of healthcare are underpinned by a focus on the biological rather than existential aspects of illness which reduces the attention awarded to the subjective experience of being unwell’ (2017, p. 176). Fear of damaging their relationship with staff also risks threatening the trusting relationship because any perceived criticism may be reacted to with ‘undue sensitivity on the part of the trusted’ (Entwistle & Quick, 2006, p. 401) and because patients may fear the consequences (Doherty & Stavropoulou, 2012). By speaking up about concerns, patients risk becoming more vulnerable because of their dependence on others to care for them.

We set out to understand the phenomenological aspects of experiencing a deterioration in condition for participants who had lived through post-surgery complications. Whilst previous research has sought to understand how patients can contribute to escalation of care and rescue processes (Rainey et al., 2015; Strickland et al., 2019), what is missing from the literature is an idiographic account, situating patients’ contribution to safety within their personal lived experiences and meaning making of these episodes of deterioration. This paper aims to address this gap by presenting a rich account of the inter- and intra-personal influences associated with individuals’ experiences of living through these episodes. We seek to facilitate an understanding of the complexity of this biopsychosocial phenomenon (Biggerstaff & Thompson, 2008) and, highlight the implications of this for designing interventions to enable involvement of patients and families in prompt recognition of and response to deterioration.

## Methods

This paper is linked to a programme of research funded by National Institute of Health Research (NIHR), looking to address the problem of failure to identify and respond to patients following abdominal surgery. This paper specifically draws on patient experience data collected as part of a larger exploratory dataset to understand current work practices as the basis for service re-design.

### Design

This qualitative study utilised a semi-structured interview design, and an interpretative phenomenological analysis (IPA) approach to explore the lived experiences (Nizza et al., 2021) of participants following abdominal surgery and an episode of clinical deterioration. IPA is typically used to provide an in-depth exploration of personal lived experiences of important life events by focusing on understanding how participants make sense of those life events, and the role identity and the self plays in this sense-making (Smith et al., 2009; Smith & Osborn, 2007). IPA is particularly useful where the research topic ‘is dynamic, contextual and subjective’ and ‘relatively under-studied’ (Smith & Osborn, 2007, p. 520).

### Sampling and Participant Recruitment

The focus and structure of IPA centres on obtaining a small number of rich experiential accounts and analysing them in detail in order to illustrate the phenomena of interest. Small sample sizes are in keeping with the commitment to idiographic depth and interpretation (Smith, 2011). We used purposive sampling as a means to select ‘information-rich cases’ (Palinkas et al., 2015), aiming to recruit six to seven individuals who had experienced the phenomenon of a deterioration in their condition post-abdominal surgery. Individuals were invited to participate from Bowel Research UK via their social media networks. Nine people expressed interest and seven participated in the study. Participants were aged between 35 and 80, all self-defined as White British and included 3 men and 4 women. All names are pseudonyms. For further details on the participants’ surgical history, see Table 1.

**Table 1.** Participants and Their Surgical History.

Name	Age	Reason for surgery	Complications
Anna	60	Removal of large bowel for cancerous polyps	Problem with anaesthetic and reaction to morphine followed by wound dehiscence^1^
Brenda	60	Hysterectomy	Inverted bowel resulted in entero-cutaneous fistula. Leak following surgery for repair of fistula
Charles	65	Bowel cancer surgery	Epidural failed to work during surgery followed by dehiscence
Diana	80	Burst diverticular	Surgery for a stoma resulted in parastomal hernia
Gordon	59	Bowel cancer surgery for temporary stoma	Stoma failed to work. Blockage resulted in another surgical procedure
Harry	74	Colorectal cancer reversal of earlier ileostomy	Paralytic ileus
Issy	35	Initially had colitis, then had surgery for stage 3 bowel cancer	In hospital for 10 days nil by mouth following changes in liver function

### Data Collection

In-depth, semi-structured telephone interviews were conducted by an experienced qualitative researcher (JW). All were audio recorded and transcribed. Interviews took place from April to June 2021 and each lasted between 60 and 90 minutes. The topic guide was developed and piloted with a member of the research study’s PPI group. The topic guide (see appendix 1) focused on participants’ experiences of recent surgery and the complications that developed post-laparotomy, care received, perceptions of safety including their ability to contribute to keeping safe. The interviewer took a narrative approach to encourage individuals to reflect on their experiences and to reconstruct events, selecting those elements highlighted as significant for their sense of self and identity. This approach is appropriate when participants are potentially vulnerable due to their ill health (Greenhalgh et al., 2005).

### Ethical Considerations

The study was approved by the Cambridge East NHS Research Ethics Committee (IRAS 270881). Prior to each interview, participants received a participant information sheet and a verbal explanation of the study, provided their informed consent in writing and agreed to the audio-recording of their interviews. Names and other potentially identifying details were removed during transcription to protect confidentiality and anonymity. To enable readers to follow the accounts of the particular individuals, each participant was assigned a pseudonym.

### Analysis

We followed the traditional steps of IPA (Smith et al., 2022), starting by ES and NM reading and re-reading each transcript in its entirety. We then conducted close line-by-line analysis of the participants concerns, experiential claims and understandings (Smith et al., 2022). Detailed notes were made on each, taking account of our individual reflections and thoughts. These initial notes were translated and abstracted into a list of experiential statements which were examined for connections and patterns across the dataset in order to create a cluster of personal experiential themes. These experiential themes were then discussed with the wider project team in order to develop and check our interpretation. The transcripts were imported into NVivo 12 in order to enable mapping of the themes and accounts. We ended up with five group experiential themes which captured idiographic depth together with shared perspectives of vulnerability (Smith et al., 2022). These five group experiential themes included: Making sense of signs and symptoms; Timelines and uncertainties; Providing legitimacy and a safety net; Dependencies on staff and; Bonds of transgression. We present an experiential account of the corpus, paying close analytical attention to participant’s words and highlighting patterns of similarity and individual difference within the narratives (Nizza et al., 2021).

## Results

Our analysis revealed one overarching group experiential theme of vulnerability which encapsulated the uncertainty of the situation all the participants found themselves in, and the nature and seriousness of their health conditions. We reveal the extent of participants’ vulnerability by detailing how they negotiated feelings of (un)safety, their reliance on family to mitigate against existential threats, their dependence on staff and the sense of transgression that arose when their expectations of care (care as imagined) did not meet the reality of their experiences (care as received).

### Making Sense of Signs and Symptoms

All the participants struggled to make sense of whether what they were experiencing was to be expected and ‘normal’ following surgery. Understandably, participants’ accounts focused on signs and symptoms and interpretation as to their significance.

Five individuals described experiencing bodily symptoms that caused them anxiety post-surgery. These symptoms included: excruciating pain, or in Diana’s words, ‘being beside myself with the pain’; dizziness and nausea and an absence of signs of normal bowel functioning after abdominal surgery. Harry’s medical expertise enabled him to identify a mismatch between his own lived experience and what he should be experiencing as part of his recovery. ‘I was experiencing a lot of nausea, a lot of bloating and no flatulence. All signs that you are starting to get back to normal functioning, none of that was happening’.

Two participants described experiencing free-floating states of feeling unwell and a general feeling of malaise following surgery. Charles describes feeling increasingly ‘worse and worse’ while Gordon’s account highlights how vague but all-encompassing his physical symptoms were. ‘Nothing was right, everything was wrong. So... so in that sense it wasn’t good, I wasn’t feeling well, I had no energy’. Others described both bodily symptoms, such as pain and thirst, along with an overwhelming feeling of ‘sinking’ or a feeling of doom as Brenda describes.

‘It was just the way that I was feeling inside, it was just impending fear and impending sense of doom that something really bad was going to happen. Physically I just felt worse than I’ve ever felt in my whole life. […] I was developing an incredible thirst to the point I was having hallucinations. About everything that I saw, cylindrically shaped was a potential drink.

Brenda’s account reveals a sense of profound existential threat. Note how her account conveys the extreme experience she faced, describing it as worse than she had ever felt in her whole life and recounting the sensation of an incredible thirst. The sense of danger is palpable yet Brenda’s words also convey her powerlessness as she was so unwell she had no choice but to wait for events to unfold. The juxtaposition of feeling passive and helpless yet aware as her condition continued to deteriorate comes to the fore through her description.

Female participants tended to talk more emotively about their experiences of cancer, surgery and complications than the male participants. This extract and Brenda’s use of the first person highlights the extreme vulnerability and fragility that was associated with the deterioration in her physical condition.

But inside I didn’t think I was going to survive this, and I remember lying on the bed looking at my phone on the locker and not having the strength to put my hand up to get the phone even, I wanted to ring my daughter. It was pretty awful really [starts to sob]. . “I said look I really feel as though I really, really need help. I just don't feel as though I am going to make it through the day’.

Brenda’s account illustrates the horror of her situation by highlighting her physical incapacity and inability to even pick up her phone to call her daughter. Her distress at this is illustrated by sobbing sounds as she actively engages with the memory of the experience. The trauma is evident as Brenda relives thinking that she was going to die. Her account highlights the long lasting effects of experiencing this vulnerability and fragility as she recounts the desperate need for others to rescue her.

In contrast, the three male participants tended to recount their experiences in the third person, as Harry’s account of his condition post-surgery reveals. ‘So, you start by feeling bloated, not passing any stool at all. So, you have gone into complete shutdown at that point’. This avoidance of ‘I’ provides some distance from lived experiences suggesting this form of talk served an important protective role for some participants sharing distressing experiential accounts.

### Timelines and Uncertainties

Participants’ perceptions of normality were often anchored to information they received pre-operatively around expected trajectories. Charles reports being initially told that he would be discharged immediately after his operation. His account highlights how his sense of normality was shaped around what he had been told should have been happening.

‘Because I don’t think that I had any understanding of how ill I was at the time or what [….] normal would look like. So I thought Jesus ... I knew they’d talked about getting home a day or two after surgery. And I remember thinking a day or two after surgery, Jesus there is no way …. I could barely stand up, let alone go home!’

Charles stresses the depth of his feeling about how unwell he felt by exclaiming Jesus in relation to the time-point when it was suggested that he should have been well enough to go home. He felt that he was in no condition to be discharged as he could barely stand up at that point. His account indicates a sense of profound incongruence between his own perception of his capabilities and what he was led to believe was expected of him. This led to feelings of uncertainty and helplessness. He was simply unable to do anything to help himself at that point, let alone be discharged.

In contrast to Charles, Anna recalled that the lack of procedural information she received served to protect her from understanding the gravity of her situation. Anna’s condition deteriorated while she was in recovery. She reflects that her understanding of the significance of this was limited by her drowsiness and her uncertainty around how her experience mapped to what was normally expected.

‘None of it made sense to me. [..] my bed was opposite a clock and I could look at the clock and think gosh am I still here! [laughs] Because I had no concept… I suppose if someone said to me you’ll be in recovery for an hour and then we will move you back to the ward, so then I looked up and thought oh I’ve been here for it looks like 7 hours and I’m still not in the ward. Then I might have been worried, but because I didn't have anything to contextualise it with, I just thought “oh I seem to be here a long time”.

Underpinning Anna’s words are a sense of disconnection and separation from the enormity of what was happening to her, almost as if she was observing herself from afar. At one level, she was aware of time but her physical condition limited her ability to engage with its meaning. This served a protective function as Anna notes ‘oh well, somebody will sort something out’.

### Providing Legitimacy and a Safety Net

References to family members peppered all the participants’ accounts, as having someone present to advocate and care for them to some extent mitigated against the feelings of powerless and extreme vulnerability. All participants reported drawing on close family reactions to sense check their conditions following their surgery. They looked to relatives for confirmation whether they ‘looked normal’ and about the significance of signs and symptoms as to whether this indicated they were ‘unwell’. Brenda ‘knew that something bad was happening’ to her and drew on her husband’s assessment as a form of legitimacy to substantiate her own sense-making. ‘I remember on the Wednesday my husband was there and a consultant came in, and he is a lovely consultant and he didn't seem unduly concerned. My husband said “look I’ve never seen in the 40 years that I have known her, there must be something going on”. .. He said, “it’s not normal, it’s not normal for her to be like this.”’ The authority Brenda attributes to her husband’s ‘situated knowledge’, based on 40 years of experience, provides Brenda with a form of embodied security. She draws legitimacy from his ‘diagnostic work’ as his intuitive insights into ‘abnormality’ become entwined with a need for action. Brenda’s account illustrates relationships between embodiment, care and affective interactions around her perceptions of deterioration.

Four participants’ accounts demonstrated how their feelings of (un)safety were mediated by relatives’ actions, in particular, performance of an important advocacy role. Harry’s sense of vulnerability and co-dependency comes through as he describes being reliant on his wife to raise concerns about his condition on his behalf. ‘I basically was feeling lousy anyway and that wasn’t being taken note of by those who were providing the direct care. It was only when my wife started to get stroppy that we actually managed to convince the registrar who agreed with our diagnosis…well her diagnosis because I think I was probably too far out of it anyway at that point. Had we not known what was going on, and I say we because I was probably less use than she was… had she not known what the symptoms I was displaying were, they probably would have gone un-noticed for a few more hours’. Harry’s reference to ‘her diagnosis’ rather than ‘our diagnosis’ illustrates the relational power imbalance he experienced on account of his acutely deteriorating physical state, which limited his agency and ability to cognitively process his symptoms. Her presence and witness to his ‘unwell state’ provides him with a much needed safety net. His awareness of the significance of this advocacy role (and what would have happened if she had not been there) comes through the account. He notes that his wife had to ‘get stroppy’ in order to be listened to, highlighting how jurisdictional challenges around diagnosis served to compound his sense of helplessness.

Linked to these feelings of helplessness of not being listened to, two participants recounted that they pleaded with their relatives to stay with them to help provide some security. Charles notes he was so anxious about his condition that he asked his wife to stay with him. ‘I couldn’t get anybody to take me seriously [..] I was feeling worse and worse and worse and that was probably the low point of the entire episode. Funny enough I was talking to the wife about it last week, and she reckoned that that went on for about 3 days to the point where one night I said I don’t want you to go home, I don’t feel well enough’. Charles’s sense of vulnerability is apparent in this extract. His feelings about the precariousness of his condition meant that he wanted and needed someone there to protect and advocate for him. By asking his wife to stay with him, he was effectively sharing his vulnerability with her.

### Dependencies on Staff

All the participants also highlighted how relationships with staff contributed to their sense-making around their condition (and its seriousness) as well as their sense of self and value as a patient. Gordon was unsettled by staff’s apparent lack of concern regarding his condition post-surgery and he describes questioning whether he was, in fact, avoiding dealing with his stoma. It was only the presence of the surgeon that helped Gordon realise that his recovery was not progressing as it should have been.

‘I didn't really know what to expect, so I wasn’t really in a position to judge, is this normal, they’re busy, there’s a job to do, maybe I’m being lazy, maybe I’m not dealing with this as well as I should be? And then I think as time went on they could see the surgeon coming in and being quite worried and then I think it did get more concerning’.

Gordon’s account illustrates a sense of powerlessness and feeling judged. He did not have the type of knowledge that would enable him to make sense of his experience. The questions he raises indicate the level of uncertainty he had as to whether he should be ‘bothering’ staff about how he felt which meant that he was unsure as to how he should behave. The arrival of the surgeon, coupled with their concern, signifies that his condition was now considered as serious. Gordon is now justified in his attempts to get attention.

Five of the seven participants described checking out their concerns about their deteriorating condition with staff. Charles describes repeatedly raising his stomach pain as a source of concern. He notes that this was attributed by staff to how recent his surgery was. ‘And you have this growing dull ache in your stomach. So I mentioned this a few times and they said just give it time, just give it time’. Charles account shows how he was concerned enough to raise his worsening stomach ache more than once. He was powerless to do anything other than report it to those who were able to help him. However, his report was dismissed by staff, who felt that his symptoms were to be expected given his recent operation. Two participants reported feeling that the nursing staff’s focus was on expected post-operative trajectories rather than listening to patients’ own individual embodied experiences. While participants reported focusing on trying to articulate a feeling or embodied sense of being unwell, this appeared to contradict staff’s foci which centred on functional knowledge. As Charles notes, ‘they just seemed to be more focused on have you moved your bowels yet? Have you […] passed urine? That seemed to be their sole concern really’.

Participants described assessing different staff for who best to connect with. Charles’s account highlights how this process of assessment involved emotional judgement as to who could provide individualised, personalised care. Charles notes the significance of one health professional who was seen to be ‘greatly in demand’ on the ward. ‘I mean some [nurses] were clearly just following the process. Others were… I’m not quite sure what they think they were doing. So you really had to get the attention of this one individual who naturally being the sort of person she was, was greatly in demand’. A sense of precariousness underpins Charles’ words as he recounts having to fight for the attention of this nurse alongside other patients.

The participants’ accounts demonstrate how patients’ states of vulnerability post-surgery heightened their dependencies on staff to have the right skills, expertise and compassion not only to listen to their concerns but also to care for them effectively. Anna describes her feeling of anxiety after being re-admitted urgently for complications post-surgery to an outlier (non-surgical) ward when she realises that there is nobody she can rely on to help her manage the bag.

‘I was so new to the stoma and nobody knew how to change the bag and of course I’m still […] learning it so the nurse and I muddled through. And then they wanted to give me a CT scan and the bag just filled up with the liquid that I needed to swallow. And I asked if this was normal and nobody knew anything! So, it was very distressing [….] because I felt that I wasn’t in the right place.’

Vulnerability and uncertainty underpin Anna’s narrative as she was left to ‘muddle through’ with staff who she perceived to lack the necessary expertise to care for her properly. A sense of existential threat and potential for harm also comes through, linked to not being in the ‘right place’. Similarly, Gordon’s experience following surgery led him to question whether the staff caring for him were knowledgeable and competent. He reports that hospital staff failed to investigate his inability to perform everyday tasks while he was in hospital. His trust in the nursing team was undermined by this dissonance between his expectations and the reality experienced.

‘You would have expected that experienced nurses would have [..] seen that this guy is not right. I was in my late 40s at the time, a fit and relatively young person, why isn’t he able to get up and why is he not feeling good?’

### Bonds of Transgression

Six participants recounted feeling let down following surgery after raising concerns about their safety. Furthermore, five of these found it hard to get staff to take their concerns seriously when they attempted to articulate their need for help. The impact of having their concerns dismissed was profoundly upsetting, creating a sense of transgression; feeling betrayed by healthcare professionals who were there to look after them.

Harry drew on his professional experience to reflect on his expectations of care and how his reality had fallen short of this care as ‘imagined’. He reports feeling that the people he should have been able to trust to take care of him were not able to fulfil this expectation. ‘My expectations were that I would be treated exactly the same as I used to teach my students how to manage an admission process. [..] One of my own masters students has just actually just completed her management care degree and was actually in charge of that hospital. I was not expecting such poor [care]’. Harry’s account demonstrates how his own medical expertise sensitised him to a lack of quality and safety in his care. It also influenced his expectations of his care and contributed to a sense of betrayal.

Brenda reports experiencing a sense of transgression and epistemic injustice when her experiential knowledge was dismissed.

‘The worst thing is, is not being believed. And I know that the nurses are overwhelmed with work and busy and all of that but I think that they should have known that I was quite on board with what was happening to me. I knew about the fistula, I knew about what had happened.’

Brenda’s account illustrates her outrage at the staff failure to believe her when she reported her symptoms. Her acknowledgement of staff busyness indicates her awareness of the context that NHS staff endure, but that nonetheless, her experience was discounted. She had prior knowledge of her condition, how it had arisen and what could be done about it. She should have been listened to.

Diana’s account highlights a lack of perceived compassion from staff which had a lasting impact on her sense of self. Diana describes a deeply unpleasant bout of sickness following her surgery when she was denied help from a nurse. ‘I felt so ill that night, and I wanted to sit up to be sick, and she said, ‘I can’t help you, I’ve got a bad back’. Having someone who was specifically in a position to care for her reject her plea for help left Diana feeling abandoned and her trust violated. Diana describes resorting to telephoning the Samaritans from her hospital bed in preference to calling for help from staff.

‘I thought that I was dying, and thought ‘I can either let myself die, yeah, or I can ask for some help’. […] But, if I’d have rung the bell, who would have come? The […] nurse who just said, ‘I can’t help you’ and was horrible to me. So, I got my phone, and I rang the Samaritans.[…] And that was a pretty painful moment’.

Diana’s account reveals her predicament. She faced a dilemma between calling for the nurse who let her down previously and calling an external organisation who were renowned for being trained to listen. In this case, Diana preferred to call and speak to those who she knew would listen to her rather than have her pleas for help ignored. This indicates how important it was to Diana to be shown that she was cared for, and that someone was there for her. The pain and distress associated with being in this compromising position comes through in Diana’s words.

Diana reports fearing repercussions if she complained about those caring for her. ‘But I didn’t [speak up] then. I didn’t. There’s something about, very much in hospital, that, if you complain, you’ll be treated worse’. Diana indicates her wariness about raising concerns about her care while still being cared for. She knew she was vulnerable. She did not complain because she was anxious whether the quality of her care would suffer as a result. She felt that staff would know she had complained and she would experience reprisals. Silence gave Diana some form of control and provided a means to protect herself from further harm.

Brenda describes an overwhelming sense of epistemic injustice associated with her care. As her health deteriorated following her post-surgery complications, Brenda became increasingly upset and angry.

‘I did try and ask the consultant for answers but he almost, I won't say normalised it that he played it down. And said, “well sometimes these things happen and there is no explanation”. And maybe that is the truth, [..] of course as time was going on I was getting more and more angry. Because [..] my health was going worse’.

Brenda’s account illustrates how she experienced a double injury; one physical and the other psychological, and feelings of abandonment as she recalls a lack of follow up emotional or psychological support.

‘it’s like a double wound, a physical wound of mutilating surgery and what it does to your body when it goes wrong. But then it is the secondary wound of realising that there is no help out there to help you come to terms with it. And all the psychological damage that it does. [..]. And I think that I am still in a very bad place as a result of it’.

Brenda indicates the strength of her feelings about the damage to her body as a result of the failure of her surgery, by referring to it as mutilating. Her account also refers to the ‘wound’ of abandonment by health professionals who she perceives have also failed her. Brenda feared further operations to rectify complications arising from the original surgery. Her account reveals the extent to which she feels emotionally and physically scarred by the whole experience. Her account highlights the legacy left for some trying to come to terms with their experiences many years later.

## Discussion

Patient safety traditionally has been understood as a professional and technical issue (Ocloo, 2010). However, when safety is explored from the perspective of patients’ sense-making, it is reframed as dynamic, contingent and contestable (Doherty & Saunders, 2013; Rhodes et al., 2015). This idiographic account of patients’ experiences of living through episodes of clinical deterioration following abdominal surgery adds to this literature and brings to the foreground, safety as embodied, visceral and relational. Our findings usefully situate how safety is intertwined with a patient’s identity and sense of self, but also how safety links to interpersonal negotiations. Following surgery, patients may experience dissonance, ‘chaos’ and ‘suffering’ when pre-surgery expectations (and trust) are violated (Doherty & Saunders, 2013). Rescue work also embodies both the routine and the emergency amidst complex patient trajectories (Mackintosh & Sandall, 2015). Whilst safety efforts tend to focus on the management of discrete episodes of deterioration, our participants brought threads of past, present and future together in their accounts, situating their illness narratives within the broader temporal and organisational context.

Vulnerability, as a core group experiential theme, underpins the participants’ narratives and substantiates previous accounts of patients’ experiences of clinical deterioration in medical and maternity settings (Rainey et al., 2015; Rance et al., 2013). Patients who underwent invasive surgery also experienced the vulnerability that arose from being unwell and at risk of death, and from their dependence on others for everyday care tasks. Additionally, they had to manage the vulnerability that emerged from their predicament of unpredictability (Misztal, 2011), that is, the uncertainty that arose immediately post-surgery but also the potential unpredictability of their future lives and how they would unfold.

Our findings additionally highlight the significance of acuity of condition, power and asymmetry. A power imbalance or functional form of asymmetry lies at the heart of the clinical relationship, which reflects the wider purpose of the institution of medicine in society (Pilnick & Dingwall, 2011). Patients are placed in a double bind by the sick role, as they are expected to use their own judgment in determining when it is appropriate to seek professional advice, but then, once in receipt of care, they are then expected to demonstrate their co-operation with legitimate expertise by deferring to the professional’s judgment (Bloor & Horobin, 1975). Any response to the professional assessment that challenges this asymmetry undermines the patient’s grounds for seeking professional medical help in the first place (Pilnick & Dingwall, 2011).

It is therefore important for approaches to improving safety to take account of patients’ part in the negotiated process of healthcare and asymmetrical power divides (Doherty & Saunders, 2013). Participants’ accounts of trying to contribute to escalation of care showed how language provides an important means of imposing order and conveying certainty. ‘Chart talk’ privileges biomedical knowledge (Mattingly, 1998) and there can be an epistemological disjuncture between the rationality associated with those clinical values portrayed as ‘valid’ and the embodied tacit knowledge of patients’ deterioration possessed by patients and relatives, found to be useful in early detection of deterioration (Odell et al., 2010).

Fricker (2007) uses the concept of ‘epistemic injustice’ to refer to when a person is unfairly harmed in her/his capacity as a knower. The participants’ accounts highlight aspects of the two related kinds of epistemic injustice, testimonial and hermeneutic. Testimonial injustice occurs when identity prejudices damage a person’s credibility unfairly (Fricker, 2007). We see how some of our participants’ embodied knowledge was dismissed or seen as less valuable on account of participants being categorised as patients, or perceived as overly anxious. Hermeneutic injustice negatively impacts on a person’s ability to articulate and give meaning to their experiences (Fricker, 2007). Several of our participants noted the lack of information and interpretative resources available to help them make sense of their subjective illness experiences. Our research builds on other studies that demonstrate that patients’ testimonials were often dismissed as emotionally unstable, irrational or unreliable due to the epistemic privilege enjoyed by both organisations and their practitioners (Carel & Kidd, 2014).

Our findings illustrate the importance of care that is both humanised and dialogic for patients. These patients were often looking for someone to support them emotionally as well as physically through their experience. Medical care is often prioritised over emotional support when patient need outstrips resources (Hope et al., 2022). Time pressures, lack of resources and a focus on task-based work practices all conspire against establishing rich communicative relationships in today’s healthcare practice (Kidd & Carel, 2017). MacDonald (2016), illustrates how small talk operates to manage the therapeutic relationship, elicit important clinical information, help patients through unpleasant procedures and manage the flow of interaction at any given point in community nursing (Macdonald, 2016). Communication is vital both to inform patients about procedures and tasks but also as a way for practitioners to glean greater understanding of the patient and their experience, thus creating an opening for the co-creation of safety in situ. As Cribb and colleagues note (2021), conversations act as a ‘reciprocal exchange in which relationships are built and sustained’ (Cribb et al., 2021, p. 2).

Lastly, our findings have shown that whilst patients and their relatives can act to scaffold the clinical system, it is imperative that the system is designed to take account of patient concerns. As McKinney and colleagues (2021) note, changes in ward culture are needed to truly hear the patient. Healthcare professionals require organisational support in order to create an environment where compassionate care is prioritised (Crawford et al., 2014). This includes supporting staff to cope with the tensions they experience between responding to patients and responding to institutional concerns of risk, accountability and resource management (Hillman, 2016).

This study has limitations. The theoretical generalisability of the current study is limited to this particular sample of self-selecting participants who were digitally active on social media and may not apply to other populations. We also recognise that retrospective accounts may be subject to recall biases (e.g. selective memory of negative events). We have learned much from patients’ idiographic accounts. Further research could usefully foreground the subjective experience of staff. Conducting an idiographic inquiry of rescue from staff's perspectives could further our understanding of safety as a relational phenomenon.

## Conclusion

The participants’ narratives of experiencing complications following abdominal surgery reveal the extent of their vulnerability. Their experiences highlight the power imbalance between patients and professionals in terms of whose knowledge counts within the hospital context and the potential for epistemic injustice to arise when patients’ concerns are ignored or dismissed. Rescue work requires an interpersonal and evaluative approach orientated towards to the value of patient and family concerns. Safety interventions should be developed with the dynamic, cognitive and sense-making nature of this safety work in mind. In terms of implications for designing interventions, this translates to the importance of acknowledging vulnerability at a personal level (psychological safety) as well as the potential benefit of relational-based strategies (e.g. co-production and advocacy).

## Note

1 A condition where a cut made during a surgical procedure separates or ruptures after it has been stitched back together.
